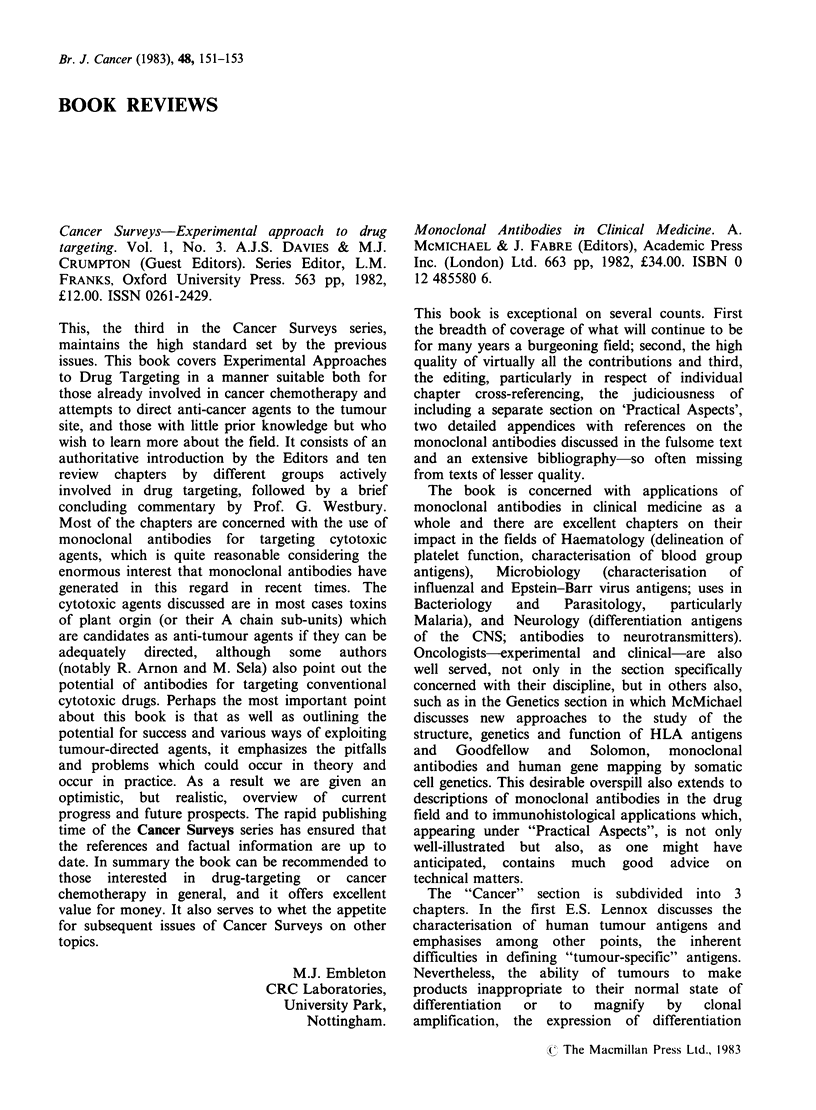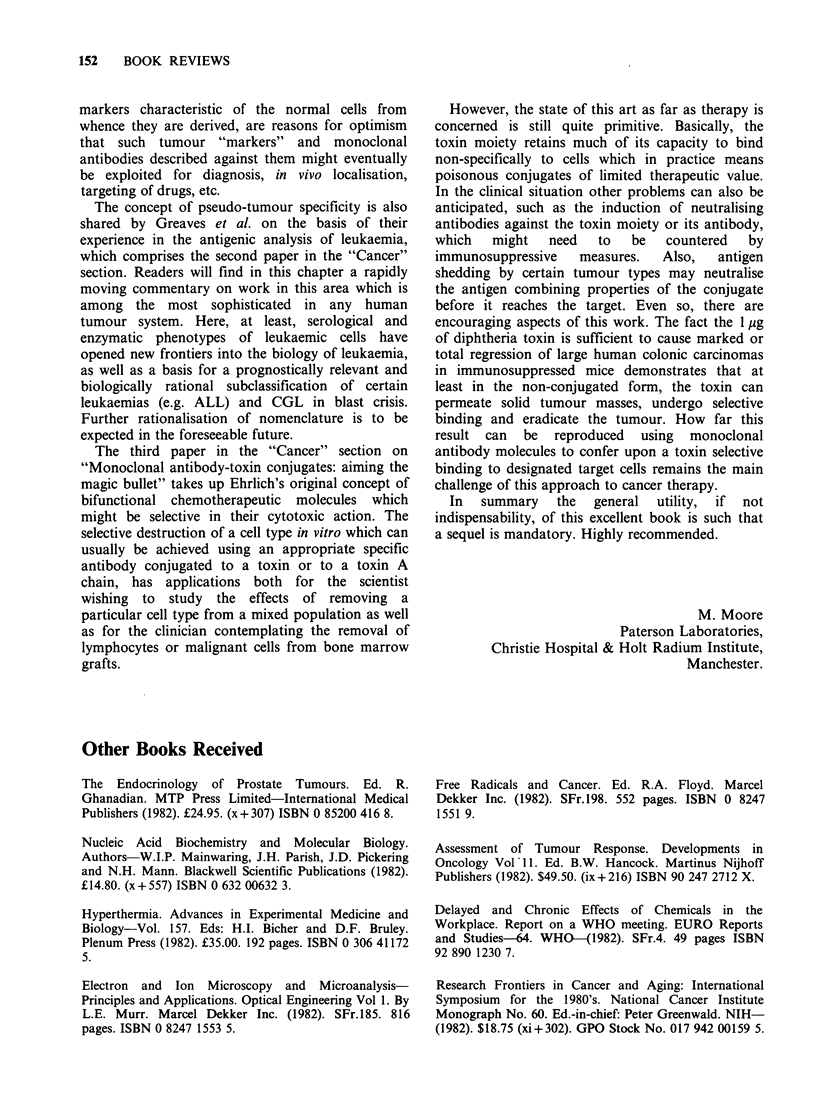# Monoclonal Antibodies in Clinical Medicine

**Published:** 1983-07

**Authors:** M. Moore


*Monoclonal Antibodies in Clinical Medicine*. A. MCMICHAEL & J. FABRE (Editors), Academic Press Inc. (London) Ltd. 663 pp, 1982, £34.00. ISBN 0 12 485580 6.

This book is exceptional on several counts. First the breadth of coverage of what will continue to be for many years a burgeoning field; second, the high quality of virtually all the contributions and third, the editing, particularly in respect of individual chapter cross-referencing, the judiciousness of including a separate section on 'Practical Aspects', two detailed appendices with references on the monoclonal antibodies discussed in the fulsome text and an extensive bibliography—so often missing from texts of lesser quality.

The book is concerned with applications of monoclonal antibodies in clinical medicine as a whole and there are excellent chapters on their impact in the fields of Haematology (delineation of platelet function, characterisation of blood group antigens), Microbiology (characterisation of influenzal and Epstein–Barr virus antigens; uses in Bacteriology and Parasitology, particularly Malaria), and Neurology (differentiation antigens of the CNS; antibodies to neurotransmitters). Oncologists—experimental and clinical—are also well served, not only in the section specifically concerned with their discipline, but in others also, such as in the Genetics section in which McMichael discusses new approaches to the study of the structure, genetics and function of HLA antigens and Goodfellow and Solomon, monoclonal antibodies and human gene mapping by somatic cell genetics. This desirable overspill also extends to descriptions of monoclonal antibodies in the drug field and to immunohistological applications which, appearing under "Practical Aspects", is not only well-illustrated but also, as one might have anticipated, contains much good advice on technical matters.

The "Cancer" section is subdivided into 3 chapters. In the first E.S. Lennox discusses the characterisation of human tumour antigens and emphasises among other points, the inherent difficulties in defining "tumour-specific" antigens. Nevertheless, the ability of tumours to make products inappropriate to their normal state of differentiation or to magnify by clonal amplification, the expression of differentiation

© The Macmillan Press Ltd., 1983

markers characteristic of the normal cells from whence they are derived, are reasons for optimism that such tumour "markers" and monoclonal antibodies described against them might eventually be exploited for diagnosis, *in vivo* localisation, targeting of drugs, etc.

The concept of pseudo-tumour specificity is also shared by Greaves *et al.* on the basis of their experience in the antigenic analysis of leukaemia, which comprises the second paper in the "Cancer" section. Readers will find in this chapter a rapidly moving commentary on work in this area which is among the most sophisticated in any human tumour system. Here, at least, serological and enzymatic phenotypes of leukaemic cells have opened new frontiers into the biology of leukaemia, as well as a basis for a prognostically relevant and biologically rational subclassification of certain leukaemias (e.g. ALL) and CGL in blast crisis. Further rationalisation of nomenclature is to be expected in the foreseeable future.

The third paper in the "Cancer" section on "Monoclonal antibody-toxin conjugates: aiming the magic bullet" takes up Ehrlich's original concept of bifunctional chemotherapeutic molecules which might be selective in their cytotoxic action. The selective destruction of a cell type *in vitro* which can usually be achieved using an appropriate specific antibody conjugated to a toxin or to a toxin A chain, has applications both for the scientist wishing to study the effects of removing a particular cell type from a mixed population as well as for the clinician contemplating the removal of lymphocytes or malignant cells from bone marrow grafts.

However, the state of this art as far as therapy is concerned is still quite primitive. Basically, the toxin moiety retains much of its capacity to bind non-specifically to cells which in practice means poisonous conjugates of limited therapeutic value. In the clinical situation other problems can also be anticipated, such as the induction of neutralising antibodies against the toxin moiety or its antibody, which might need to be countered by immunosuppressive measures. Also, antigen shedding by certain tumour types may neutralise the antigen combining properties of the conjugate before it reaches the target. Even so, there are encouraging aspects of this work. The fact the 1 $\mu$g of diphtheria toxin is sufficient to cause marked or total regression of large human colonic carcinomas in immunosuppressed mice demonstrates that at least in the non-conjugated form, the toxin can permeate solid tumour masses, undergo selective binding and eradicate the tumour. How far this result can be reproduced using monoclonal antibody molecules to confer upon a toxin selective binding to designated target cells remains the main challenge of this approach to cancer therapy.

In summary the general utility, if not indispensability, of this excellent book is such that a sequel is mandatory. Highly recommended.

M. Moore
Paterson Laboratories,
Christie Hospital & Holt Radium Institute,
Manchester.